# Characterisation of COVID-19 deaths by vaccination types and status in Malaysia between February and September 2021

**DOI:** 10.1016/j.lanwpc.2021.100354

**Published:** 2022-01-01

**Authors:** Nur Asheila Abdul Taib, Dhesi Baha Raja, Alvin Kuo Jing Teo, Adeeba Kamarulzaman, Timothy William, Arvinder-Singh HS, Siti Aisah Mokhtar, Choo-Yee Ting, Wei Aun Yap, Michelle Chan Yoon Kim, Lidwina Edwin Amir

**Affiliations:** a AINQA Health Sdn. Bhd., Malaysia; b Saw Swee Hock School of Public Health, National University of Singapore, National University Health System, Singapore; c Department of Medicine, Faculty of Medicine, University of Malaya, Kuala Lumpur, Malaysia; d Gleneagles Hospital, Kota Kinabalu, Sabah, Malaysia; e Infectious Diseases Society Kota Kinabalu Sabah-Menzies School of Research Clinical Research Unit, Kota Kinabalu, Sabah, Malaysia; f Queen Elizabeth Hospital-Clinical Research Centre, Ministry of Health, Kota Kinabalu, Malaysia; g Department of Community Health, Faculty of Medicine, Universiti Kebangsaan Malaysia, Malaysia; h Faculty of Medicine and Health Sciences, Universiti Putra Malaysia, Malaysia; i Faculty of Computing and Informatics, Multimedia University, Malaysia; j Quanticlear Solutions Sdn. Bhd., Malaysia

We report COVID-19 deaths by vaccine types (inactivated whole-virion SARS-CoV-2 [hereinafter inactivated vaccine], BNT162b2, and ChAdOx1 vaccines), and vaccination status in Malaysia and further stratified the analysis by the presence of comorbidities. We used line list data on COVID-19 deaths until 14 September 2021, made available publicly by the Ministry of Health Malaysia.^1^ We limited the analysis from 24 February 2021 onwards, after COVID-19 vaccines were introduced. For all vaccine types, we defined individuals to be fully vaccinated 14 days after the final dose. We considered individuals who received only the first dose or died within 14 days after the final dose as partially vaccinated. We calculated the age-standardised mortality rate per 100,000 population (ASMR) by the direct method of standardisation using the Malaysian population^2^ and the World Health Organization (WHO) standard population as reference^3^ and their corresponding 95% confidence interval.

Between 24 February 2021 and 14 September 2021, 20,823 COVID-19 deaths were recorded (Supplementary Table 1). The median age of people who died of COVID-19 was 61.0 years (interquartile range: 49–72). Most deaths (4,946/20,823 24%) occurred in the 60-69 years group (Supplementary Table 2). Males outnumbered females with a ratio of 1.33:1. More deaths occurred among Malaysians (87.5%) and those with comorbidities (72.3%).

In comparison with vaccinated individuals, we observed higher mortality rates among those who were unvaccinated. The weekly ASMRs of fully vaccinated individuals were consistently lower than the partially vaccinated group throughout the study (Figure 1A). However, we found that individuals fully vaccinated with the inactivated vaccine had higher ASMRs than those who were partially vaccinated with the same vaccine after 25^th^ August 2021. Nevertheless, we hypothesised that there was no difference between the mean ASMRs of those who were fully and partially vaccinated with the inactivated vaccine during the period of interest. We tested the hypothesis and the mean ASMRs did not differ significantly between the groups (Student's t-test, t(4)=-0.68, p>0.05).

**Figure 1:**
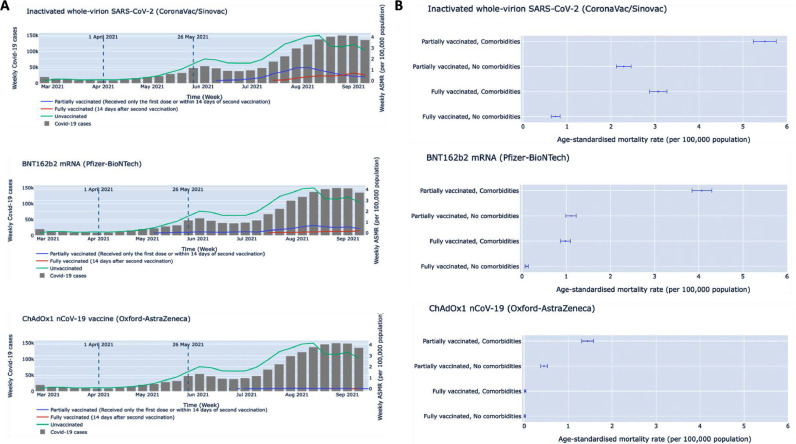
COVID-19 age-standardised mortality rates by vaccine types and vaccination status in Malaysia, 24 February to 14 September 2021. (A) Time series plot of age-standardised mortality rates for unvaccinated, partially vaccinated, and fully vaccinated individuals by vaccine types. The line graphs represent the age-standardised mortality rates. The bar charts represent weekly COVID-19 positive cases. The dashed line represents the date when the Beta variant (1 April 2021) and Delta variant (26 May 2021) was first detected in Malaysia. (B) Interval plot of age-standardised mortality rates per 100000 population with 95% confidence interval by vaccine types, vaccination status, and presence of comorbidities.

The total ASMRs for the unvaccinated group (47.5 per 100,000 population) were 43.2 times and 12.5 times higher than the ASMR of individuals fully vaccinated with BNT162b2 (1.1 per 100,000) and inactivated vaccines (3.8 per 100,000), respectively.

Among those vaccinated (Figure 1B and Supplementary Table 3), the ASMRs of partially vaccinated individuals with comorbidities were higher than those without comorbidities across all vaccine types (BNT162b2: 3.6 times; inactivated vaccine: 2.4 times; and ChAdOx1: 3.1 times). The trends were similar for fully vaccinated individuals—the ASMRs for those with comorbidities were 8.9 times, 4.1 times, and 1.5 times higher than those without comorbidities among recipients of BNT162b2, inactivated vaccine, and ChAdOx1 vaccine, respectively. However, the difference in ASMRs of individuals fully vaccinated with the ChAdOx1 vaccine was small (by two deaths). Therefore, we could not conclude the effect of comorbidities on the mortality rates of ChAdOx1 vaccine recipients in this study.

While we did not analyse immunological data in this study, the higher mortality rate among recipients of inactivated vaccines calls for close monitoring of breakthrough infections and deaths by vaccine types and further investigations into the recipients’ immunological profiles. Evidence from Hong Kong indicated differing concentrations of neutralising antibodies between the inactivated (lower) and BNT162b2 vaccine recipients (higher),^4^ highlighting potential differences in vaccine effectiveness. Our study findings also concur with the recent recommendations by the WHO to offer a third dose to persons aged ≥60 who received inactivated vaccines.^5^

In this study, we could not further describe the risk of death by the types of comorbidities. Nevertheless, other studies in Malaysia have reported that persons with diabetes, hypertension, kidney diseases, heart diseases, and cancer were at higher risk of COVID-19-related deaths.^6^^,^^7^ We could not infer vaccine effectiveness because we lack detailed information regarding those who received the vaccine but did not get infected/die. Vaccine allocation was not entirely random. Front line workers and older adults with comorbidities were prioritised for the BNT162b2 vaccine at first. However, subsequent allocation hinged predominantly on available stocks and supplies (Supplementary Table 4). Due to the concerns regarding the safety of the ChAdOx1 vaccine when it was introduced, Malaysia excluded it from the national vaccination programme and allowed voluntary opt-in through an online system. We opined that the recipients of the ChAdOx1 vaccine were likely to be younger and socioeconomically advantaged. Therefore, the outcome might be biased due to the populations’ lower risk of severe illness and death.

This study highlighted that COVID-19 mortalities were associated with vaccination status, age, and comorbidities. The mortality rate among people who were vaccinated was consistently lower than those who were unvaccinated. Among vaccinated individuals, the mortality rate of those who received inactivated vaccines was higher than the recipients of the BNT162b2 and ChAdOx1 vaccines. Vaccination coverage, monitoring breakthrough hospitalisations and deaths, safeguarding hospital capacity, and evidence-based booster strategy is critical to prevent COVID-19 mortalities.

## Author contributions

DBR, MCYK, LEA, WAY, NAAT, CYT, and AKJT conceptualised and designed the study. NAAT and AKJT verified and cleaned the data. All authors had full access to the data. NAAT, AHS, SAM, and AKJT performed the analysis. CYT, WAY, AK, TW, AHS, SAM, and AKJT interpreted the results. NAAT, AKJT, AHS, and SAM drafted the manuscript. AKJT and NAAT prepared all tables and figures. All authors contributed to the final version of the manuscript, read and approved the manuscript, and declared full responsibility for the findings presented.

## Declaration of interests

Author(s) declared no competing interest.

## Appendix Supplementary materials

Image, application 1
