# Supplementary material for: Characterisation of COVID-19 deaths by vaccination types and status in Malaysia between February and September 2021
Source: Lancet Reg Health West Pac. 2022 Jan 1;18:100354. doi: 10.1016/j.lanwpc.2021.100354 (PMC8720239; doi:10.1016/j.lanwpc.2021.100354)
Supplement: Supplementary file 1 [file mmc1.docx]

**Supplementary Materials**

**Supplementary Table 1: Characteristics and vaccination status of COVID-19 deaths in Malaysia, 24 February to 14 September 2021.**

|  | COVID-19 deaths (n=20823) | |
| --- | --- | --- |
|  | Median (IQR) | |
| Age, in years | 61.0 (49–72) | |
|  | Frequency | % |
| Sex |  |  |
| Male | 11900 | 57.1 |
| Female | 8923 | 42.9 |
| Comorbidities |  |  |
| Yes | 15065 | 72.3 |
| No | 5758 | 27.7 |
| Nationalities |  |  |
| Malaysian | 18220 | 87.5 |
| Non-Malaysian | 2603 | 12.5 |
| Vaccination |  |  |
| Yes | 6093 | 29.3 |
| BNT162b2 vaccine (Pfizer-BioNTech) | 1919 | 31.5* |
| Inactivated whole-virion SARS-CoV-2 vaccine (CoronaVac/Sinovac) | 3577 | 58.7* |
| ChAdOx1 vaccine (Oxford-AstraZeneca) | 582 | 9.6* |
| Others | 15 | 0.2* |
| No | 14730 | 70.7 |
| Vaccination status |  |  |
| Partially vaccinated |  |  |
| Only dose 1 | 4231 | 69.4 |
| Within 14 days of the final dose | 402 | 6.6 |
| Fully vaccinated (14 days after the final dose) | 1460 | 24.0 |
| Vaccine type and vaccination status |  |  |
| BNT162b2 vaccine (Pfizer-BioNTech) |  |  |
| Partially vaccinated | 1602 | 83.5 |
| Fully vaccinated | 317 | 16.5 |
| Inactivated whole-virion SARS-CoV-2 vaccine (CoronaVac/Sinovac) |  |  |
| Partially vaccinated | 2444 | 68.3 |
| Fully vaccinated | 1133 | 31.7 |
| ChAdOx1 vaccine (Oxford-AstraZeneca) |  |  |
| Partially vaccinated | 572 | 98.3 |
| Fully vaccinated | 10 | 1.7 |

IQR; interquartile range

**Percentage of those who received COVID-19 vaccines*

**Supplementary Table 2: Age-specific death rates per 100000 population, 24 February 2021 - 14 September 2021**

| Age group (years) |  | Age-specific death rates per 100000 population | | | | | |
| --- | --- | --- | --- | --- | --- | --- | --- |
|  | Unvaccinated | Partially vaccinated | | | Fully vaccinated | | |
|  |  | BNT162b2 vaccine | Inactivated whole-virion SARS-CoV-2 vaccine | ChAdOx1 vaccine | BNT162b2 vaccine | Inactivated whole-virion SARS-CoV-2 vaccine | ChAdOx1 vaccine |
| 15-19 | 2.267 | 0.091 | 0.000 | 0.091 | 0.000 | 0.000 | 0.000 |
| 20-24 | 3.612 | 0.313 | 0.278 | 0.035 | 0.035 | 0.069 | 0.000 |
| 25-29 | 10.772 | 0.412 | 1.235 | 0.137 | 0.000 | 0.137 | 0.000 |
| 30-34 | 18.511 | 1.443 | 2.323 | 0.422 | 0.000 | 0.352 | 0.000 |
| 35-39 | 29.008 | 2.226 | 4.050 | 0.876 | 0.182 | 0.292 | 0.000 |
| 40-44 | 48.719 | 3.660 | 7.685 | 1.052 | 0.320 | 0.961 | 0.000 |
| 45-49 | 69.807 | 5.005 | 11.959 | 1.159 | 0.580 | 2.160 | 0.053 |
| 50-54 | 82.684 | 12.241 | 16.340 | 1.732 | 0.751 | 3.984 | 0.000 |
| 55-59 | 100.728 | 15.012 | 23.070 | 2.599 | 1.365 | 7.538 | 0.000 |
| 60-64 | 132.288 | 16.861 | 26.079 | 7.564 | 3.624 | 12.528 | 0.236 |
| 65-69 | 172.031 | 20.213 | 32.736 | 10.766 | 4.065 | 25.047 | 0.220 |
| 70-74 | 225.197 | 29.134 | 37.953 | 12.913 | 9.291 | 29.134 | 0.157 |
| 75-79 | 294.378 | 30.739 | 37.109 | 11.908 | 11.354 | 33.232 | 0.000 |
| 80-84 | 415.676 | 40.511 | 46.235 | 20.696 | 14.531 | 46.675 | 0.440 |
| 85+ | 476.348 | 40.704 | 44.554 | 26.953 | 23.652 | 35.204 | 1.100 |

**Supplementary Table 3: Number of COVID-19 deaths and age-standardised mortality rate by vaccine types, vaccination, and comorbidities**

|  | Number of deaths recorded (age-standardised mortality rate per 100,000 population and the 95% confidence interval) by the presence of comorbidities | | |
| --- | --- | --- | --- |
| Vaccine types, vaccination status, and comorbidities | 24 February –14 September 2021 | 1 April –25 May 2021  (Beta variant) | 26 May –14 September 2021  (Beta and Delta variant) |
| Inactivated whole-virion SARS-CoV-2 vaccine (CoronaVac/Sinovac) | | | |
| Partially vaccinated | 2444 (7.79, 95% CI 7.478-8.095) | 2(0.01, 95% CI 0-0.015) | 2442 (7.78, 95% CI 7.471-8.089) |
| Comorbidities |  |  |  |
| Yes | 1714 (5.50, 95% CI 5.236-5.756) | 1(0.003, 95% CI 0-0.010) | 1713 (5.49, 95% CI 5.232-5.752) |
| No | 730 (2.29, 95% CI 2.124-2.457) | 1(0.003, 95% CI 0-0.009 | 729 (2.29, 95% CI 2.121-2.454) |
| Fully vaccinated | 1133 (3.82, 95% CI 3.596-4.040) | 0 | 1133 (3.82, 95% CI 3.596-4.040) |
| Comorbidities |  |  |  |
| Yes | 910 (3.07, 95% CI 2.874-3.273) | 0 | 910 (3.07, 95% CI 2.874-3.272) |
| No | 223 (0.74, 95% CI 0.647-0.842) | 0 | 223 (0.74, 95% CI 0.647-0.842) |
| BNT162b2 vaccine (Pfizer-BioNTech) | | | |
| Partially vaccinated | 1602 (5.18, 95% CI 4.927-5.434) | 42(0.15, 95% CI 0.103-0.193) | 1559 (5.03, 95% CI 4.780-5.280) |
| Comorbidities |  |  |  |
| Yes | 1257 (4.07, 95% CI 3.847 -4.297) | 38(0.13, 95% CI 0.091-0.176) | 1218 (3.94, 95% CI 3.715-4.157) |
| No | 345 (1.11, 95% CI 0.991 -1.225) | 4(0.01, 95% CI 0 -0.028) | 341 (1.09, 95% CI 0.978-1.210) |
| Fully vaccinated | 317 (1.09, 95% CI 0.969-1.209) | 0 | 317 (1.09, 95% CI 0.969-1.209) |
| Comorbidities |  |  |  |
| Yes | 285 (0.98, 95% CI 0.867 -1.094) | 0 | 285 (0.98, 95% CI 0.867-1.094) |
| No | 32 (0.11, 95% CI 0.071-0.146) | 0 | 32 (0.11, 95% CI 0.071-0.146) |
| ChAdOx1 vaccine (AstraZeneca) | | | |
| Partially vaccinated | 572 (1.89, 95% CI 1.736-2.046) | 0 | 572 (1.89, 95% CI 1.736-2.046) |
| Comorbidities |  |  |  |
| Yes | 434 (1.44, 95% CI 1.305-1.576) | 0 | 434 (1.44, 95% CI 1.305-1.576) |
| No | 138 (0.45, 95% CI 0.376-0.526) | 0 | 138 (0.45, 95% CI 0.376-0.526) |
| Fully vaccinated | 10 (0.03, 95% CI 0.012-0.053) | 0 | 10 (0.03, 95% CI 0.012-0.053) |
| Comorbidities |  |  |  |
| Yes | 6 (0.02, 95% CI 0.004-0.037) | 0 | 6 (0.02, 95% CI 0.004-0.037) |
| No | 4 (0.01, 95% CI 0-0.025) | 0 | 4 (0.01, 95% CI 0-0.025) |

**Supplementary Table 4: Vaccine allocation and administration by month**

| Vaccine type and allocation (%) by month | Feb | Mar | April | May | June | July | Aug | Sep |
| --- | --- | --- | --- | --- | --- | --- | --- | --- |
| Inactivated whole-virion SARS-CoV-2 vaccine | 0% | 1.2% | 15.3% | 24.2% | 52.6% | 59.7% | 43.3% | 24.6% |
| BNT162b2 vaccine | 100% | 98.8% | 84.7% | 61.1% | 35.8% | 34.1% | 48.7% | 66.5% |
| ChAdOx1 vaccine | 0% | 0% | 0% | 14.7% | 11.6% | 6.3% | 7.8% | 7.7% |
